# Development of a 3D, networked multi-user virtual reality environment for home therapy after stroke

**DOI:** 10.1186/s12984-018-0429-0

**Published:** 2018-10-05

**Authors:** Kristen M Triandafilou, Daria Tsoupikova, Alexander J Barry, Kelly N Thielbar, Nikolay Stoykov, Derek G Kamper

**Affiliations:** 1 Shirley Ryan AbilityLab, Arms + Hands Lab, Chicago, IL USA; 2 School of Design, University of Illinois at Chicago (UIC), Chicago, IL USA; 3 UNC/NC State Joint Department of Biomedical Engineering, University of North Carolina at Chapel Hill, Chapel Hill, NC USA; 4 Closed-Loop Engineering for Advanced Rehabilitation Research Core, University of North Carolina at Chapel Hill, Chapel Hill, NC USA

**Keywords:** Stroke, Rehabilitation, Virtual reality, Serious games, Upper extremity

## Abstract

**Background:**

Impairment of upper extremity function is a common outcome following stroke, to the detriment of lifestyle and employment opportunities. Yet, access to treatment may be limited due to geographical and transportation constraints, especially for those living in rural areas. While stroke rates are higher in these areas, stroke survivors in these regions of the country have substantially less access to clinical therapy. Home therapy could offer an important alternative to clinical treatment, but the inherent isolation and the monotony of self-directed training can greatly reduce compliance.

**Methods:**

We developed a 3D, networked multi-user Virtual Environment for Rehabilitative Gaming Exercises (VERGE) system for home therapy. Within this environment, stroke survivors can interact with therapists and/or fellow stroke survivors in the same virtual space even though they may be physically remote. Each user’s own movement controls an avatar through kinematic measurements made with a low-cost, Kinect™ device. The system was explicitly designed to train movements important to rehabilitation and to provide real-time feedback of performance to users and clinicians. To obtain user feedback about the system, 15 stroke survivors with chronic upper extremity hemiparesis participated in a multisession pilot evaluation study, consisting of a three-week intervention in a laboratory setting. For each week, the participant performed three one-hour training sessions with one of three modalities: 1) VERGE system, 2) an existing virtual reality environment based on Alice in Wonderland (AWVR), or 3) a home exercise program (HEP).

**Results:**

Over 85% of the subjects found the VERGE system to be an effective means of promoting repetitive practice of arm movement. Arm displacement averaged 350 m for each VERGE training session. Arm displacement was not significantly less when using VERGE than when using AWVR or HEP. Participants were split on preference for VERGE, AWVR or HEP. Importantly, almost all subjects indicated a willingness to perform the training for at least 2–3 days per week at home.

**Conclusions:**

Multi-user VR environments hold promise for home therapy, although the importance of reducing complexity of operation for the user in the VR system must be emphasized. A modified version of the VERGE system is currently being used in a home therapy study.

**Electronic supplementary material:**

The online version of this article (10.1186/s12984-018-0429-0) contains supplementary material, which is available to authorized users.

## Background

Chronic upper extremity impairment is all too common among the more than 7 million stroke survivors in the U.S. [1]. These impairments have disabling effects on all facets of life, including self-care, employment, and leisure activities. Repetitive practice of movement, such as arm movement, is thought to improve outcomes for stroke survivors [2–4], but access to the clinic for therapy is often limited by geography or lack of transportation. While almost 50 million Americans live in rural areas, 90% of physical and occupational therapists live in major urban areas [5]. Per capita ratios of therapists to overall population are 50% larger in urban as compared to rural regions of the country [6]. Rates of stroke in these rural areas, however, exceed those of major urban areas [7–9]. Thus, a large number of stroke survivors have limited access to skilled treatment. Data from 21 states found that only 30% of stroke survivors received outpatient rehabilitation, a much lower percentage than that recommended by clinical practice guidelines [10]. Declines seen following discharge from inpatient rehabilitation are undoubtedly exacerbated by limited access to clinical therapy [11].

Disparity in quality of care has been recognized in the acute treatment of stroke for a number of years. This situation has led to the development of telemedicine to extend expert care to individuals during the initial hours and days following the stroke, advance site-independent treatment, and create models of care in rural areas [12–14]. Therapy options after this acute period, however, generally remain limited for stroke survivors in rural areas. Akin to the telemedicine efforts, telerehabilitation treatments have been proposed. However, telerehabilitation interactions are typically limited to off-line monitoring by the therapist [8, 9, 15], phone calls between a therapist and client [16, 17], or videoconferencing [18–20]. While systems allowing more direct interaction have been proposed, the hardware cost and complexity limit applicability for home-based therapy [21–23]. Hence, the therapist is relegated to the role of observer and the intimacy of a clinical therapy session is lost. Therapy options are substantially restricted, as is the available feedback.

Recently, multiple investigators have been exploring means of improving home-based therapy through the development of systems or serious games which permit multiple, simultaneous users [24–30]. These efforts have proposed the inclusion of multiple users as a means to overcome resistance to home-based therapy that may result due to isolation or lack of engagement. Indeed, studies have observed a preference for multi-user vs, single-user therapy when utilizing these systems [26, 29]. However, these systems have largely been limited to control of a one-dimensional or two-dimensional space and both users remain in the same physical location (e.g., side by side). One team of researchers did develop a framework for supporting distant users (such as a therapist in the hospital and a stroke survivor in their home), but game control was limited to one or two dimensions [31, 32].

Here, we describe the development of a fully three-dimensional (3D) virtual reality environment (VRE) for home-based therapy in which multiple, remote users can interact in real time. In this Virtual Environment for Rehabilitative Gaming Exercises (VERGE) system [33], movement of the user is mapped to corresponding movement of an avatar to foster a sense of presence in and engagement with the VRE. The 3D environment encompasses aspects of clinical therapy, such as transport of objects or movement of the hand into specified regions of the upper extremity workspace. Although the importance of 3D movements in VR environments is a topic of debate [34, 35], movements tested in environments with lesser degrees-of-freedom (DOF) are often very limited and dictated by a one DOF robot. These movements differ substantially from the types of movements normally seen in 3D reaching movements [4, 36]. The network architecture of the system allows users to be located remotely from each other, such as a stroke survivor in their home, a therapist in a clinic, or a stroke survivor’s friend or relative living in another city or state. The virtual nature of the environment allows even very limited movements in the physical world to have successful functional outcomes in the virtual world, thereby offering a sense of accomplishment and motivation for successive attempts. Additionally, task difficulty can easily be modified in order to maintain the proper level of challenge, which is important for motor learning in general [37] and rehabilitation in particular [38].

We developed and performed preliminary testing of the VERGE system to gauge user response in comparison to two other therapy modalities that could be used for home therapy: an existing virtual reality system based on the Alice in Wonderland story (AWVR) [39] and a home exercise program (HEP). Fifteen stroke survivors completed three, one-hour therapy sessions per week with each of the three therapy modalities (9 sessions total). We hypothesized that the use of the VERGE system would not decrease the amount of arm movement promoted, in comparison with the AWVR and HEP modalities. We further expected that users’ self-described engagement would be greatest for the VERGE system due to the presence of a partner.

## Methods

### VERGE System

#### Architecture

At its core, VERGE consists of a 3D VRE in which avatars interact with virtual objects. To date, we have created two such VREs, one depicting a dining room and the other a kitchen. The scenes were created in Maya (Autodesk Inc., San Rafael, CA) and imported into Unity 3D (Unity 4.5, Unity Technologies, San Francisco, CA), the software platform controlling VERGE. The VREs are rich in detail in order to provide depth cues [40]. Thus, depth can be conveyed without the need for stereovision, such as that provided by head mounted displays (HMDs). We have found that HMDs can be difficult for stroke survivors to use due to the limited field-of-view and, especially, involuntary coupling between neck and arm motion [41, 42]. The latter may lead to complications with moving the arm while keeping the head steady.

**Fig. 1:**
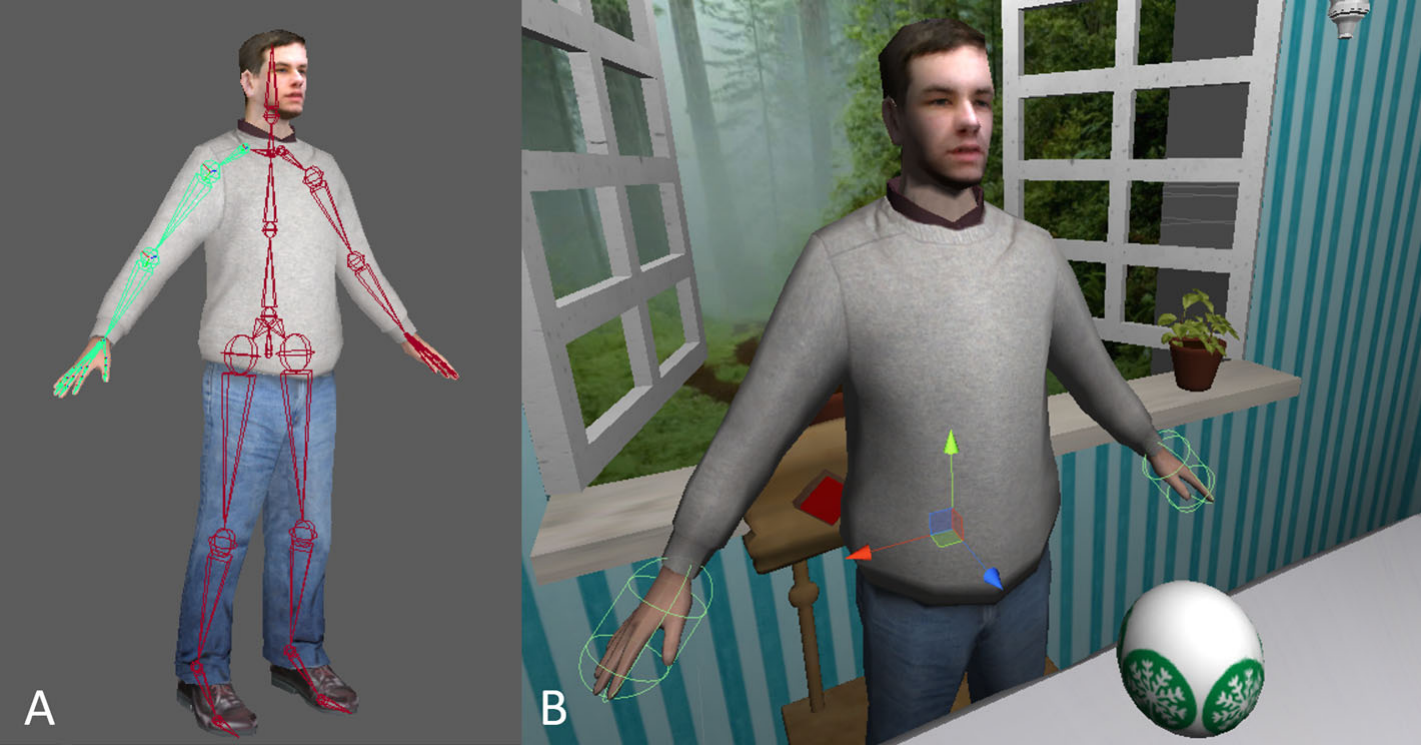
Avatar kinematics. **a** 3D model of the avatar with underlying custom skeleton as displayed in Maya. **b** Avatar imported into custom scene in Unity. Visible coordinate frame indicates location of right hip joint. Ellipsoid encompassing hands represents the contact regions for the hand colliders

The avatars were created from a custom skeleton in Maya (Autodesk Inc., San Rafael, CA), which was rigged to an existing mesh of the “casual young man” 3D model, purchased and modified for our project (Fig. 1). We created the custom skeleton to match the topology of the existing character while corresponding to the skeletal joint naming convention in Unity 3D. The skeleton (and thus avatar) is animated according to joint angle data captured with a Kinect™ I optical tracker (Microsoft Corp., Redmont, WA). The 3D motion data from the Kinect™ are transmitted to the Unity code through UDP to drive the movement of the avatar in the virtual environment.

The VERGE system employs a central server interacting with peripheral client computers, one for each user. The server receives information from the client computers and controls updating of the scene so that the appropriate view of the scene is shown on each client computer through TCP/IP network architecture. We implemented communication between the client computers through custom libraries in C# (Microsoft Visual Studio). We used two multi-user network models: an authoritative server (server performs all physics calculations) for when more than one user could interact with virtual objects simultaneously and a non-authoritative server (local computation of physics) for when only one user at a time could interact with the virtual environment.

#### Exercises

We have created three exercises (*Ball Bump, Food Fight, and Trajectory Trace*) employing Unity 3D and C#. These exercise were designed to encourage upper extremity movement, particularly to areas of the workspace that are often difficult to reach, such as those requiring raising the arm and reaching away from the body [43]. For each exercise, VERGE provides a first-person view of the virtual scene, as through the eyes of the avatar, to each user in accordance with previous studies [44]. This helps to establish a sense of presence for the user in the scene. The server displays a third-person view of the VRE.

**Fig. 2:**
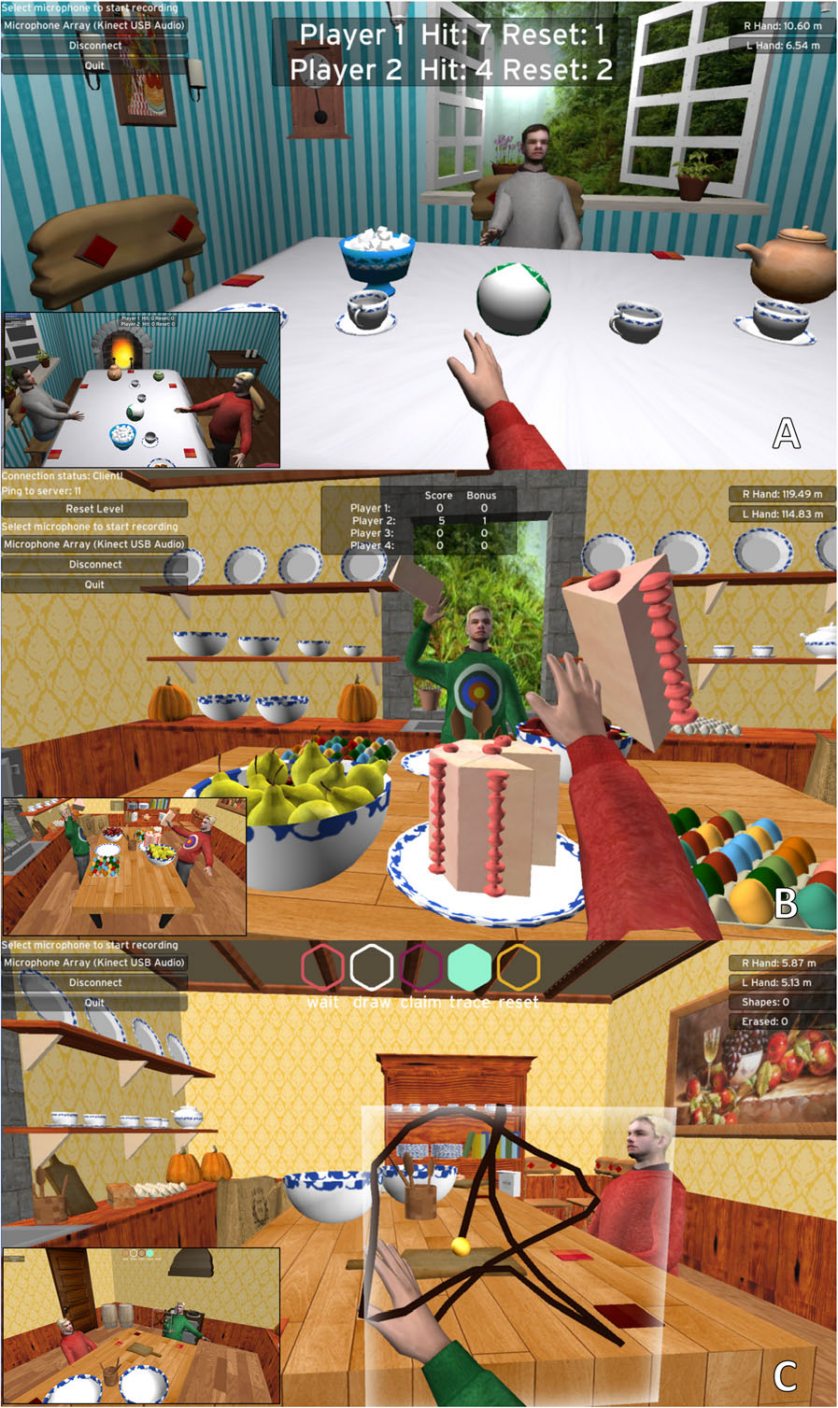
Three exercises developed for the VERGE system. First-person view of the user (client) is shown for each exercise. The inset image shows the corresponding third-person server view. **a** *Ball Bump*. Users pass a ball back and forth across the Table. **b** *Food Fight. Players pick up food items and throw them at each other.* **c** *Trajectory Trace. One player draws a trajectory in space; another player must retrace the trajectory to erase it*

*Ball Bump* is played on the table of the dining room VRE created in Maya (Fig. 2a). The goal is to hit a ball back and forth across the table, while avoiding the objects on the table. Contact between the ball and the avatar hand produces a collision that redirects the ball according to the Unity physics engine. Similarly, collisions between the ball and other objects redirect the ball. The ball will fall off the table if a fellow participant misses making contact with it and /or if the user hits the ball in the wrong direction. Pressing a red napkin, located to the side, produces a new ball. Thus, participants are encouraged to reach away from their bodies, especially to contact the napkin or to free the ball when it becomes stuck behind an object. This can be a collaborative exercise, in which the participants try to make as many successful passes as possible before the ball falls off the table, or a competitive game in which each player tries to hit the ball past the other player. Prior studies have shown that different users will have different preferences for competitive or collaborative exercises [30].

The *Food Fight* game takes place in the custom kitchen VRE created in Maya. Participants grab different food items and throw them at other avatars (Fig. 2b). The user “grasps” an object by placing the avatar’s hand in close proximity and clicking a button on a wireless optical pen mouse (2.4GHz Wireless Optical Pen Mouse Adjustable 500/1000DPI, Docooler) with either hand (stroke survivors typically operate the pen mouse with the ipsilesional hand). The user releases the object by operating another button on the pen mouse; the object’s trajectory is determined by the object’s velocity vector at the time of release. Once all of the food items have been thrown (e.g., cake, eggs, and fruit), the user can reset the food items by clicking on the reset button to continue play. We integrated mesh deformations and special effects for food items colliding with other objects. Eggs splatter and appear as yolks, the cake morphs into a pile of crumbs, and the pears and apples deform into broken fruit. Scenes are updated by the central server in authoritative mode.

In the *Trajectory Trace* exercise (Fig. 2c) one participant draws a 3D trajectory in the air. This trajectory is then passed to another participant who attempts to erase it by retracing. The state of the game (Draw, Claim, Trace, or Reset), as well as the initiation and termination of drawing the curve, is controlled by touching a button (located on the avatar’s chest) with the less affected hand. The trajectory is anchored to the avatar, such that the user must reach with the arm rather than using the trunk to move the hand to the trajectory, similar to a previous study [45]. The partner (such as a therapist) can specify to which part of the workspace the other player (e.g., a stroke survivor) should practice reaching, by drawing the trajectory in that region. To help with depth perception, a translucent 3D cube outlines the boundaries of the drawn 3D shape. Since the trajectory must be retraced in the same direction in which it was created, we display a yellow sphere to indicate the current starting point. As the user successfully traces the trajectory and it disappears, the 3D depth cube adjusts to match the volume of the remaining trajectory in real time.

### Pilot Study

A pilot study was performed to examine the feasibility of the system for use by stroke survivors. Especially, we wanted to test the amount of movement of the impaired arm, particularly to certain regions of the upper extremity workspace. Specifically, we wanted to determine whether employment of the VERGE system reduced arm movement in comparison with other potential home treatment options, namely the AWVR and HEP. We also wanted to compare user engagement across the three modalities.

#### Participants

Fifteen stroke survivors (10 male/ 5 female) in the chronic stage of recovery participated in this study. Subjects were at least 2 years post-stroke (mean of 17.4 years) and ranged in age from 33 to 81 years. Subjects had upper extremity hemiparesis, rated as Stage 3 - Stage 5 on the Stage of Arm subsection of the Chedoke-McMaster Stroke Assessment Scale [46] by a trained therapist. Subjects had no known orthopedic disease, significant visual deficits, contracture or pain (self-reported pain less than 6 on a 10-point scale) in the arm that would have hampered performing the experiments. Northwestern University’s Institutional Review Board (Chicago, IL) approved the study design and each participant signed an informed consent before study enrollment.

#### Intervention

Each participant took part in a three-week intervention study consisting of 9 one-hour training sessions in a laboratory setting over 3 weeks. During each week, the participant was involved in one of three therapy modalities 1) VERGE, 2) HEP or 3) AWVR. The order of the therapy was randomized for each participant, and all participants took part in all three therapies.

The VERGE therapy utilized the three different exercises previously described*. Ball Bump* was always the first exercise introduced to the participant as it was deemed the simplest to understand, and thus provided a straightforward means for learning to control the avatar in the VRE. *Trajectory Trace* and *Food Fight* were then played, in that order. All exercises for the VERGE therapy were performed with the arm unsupported. Participants performed each exercise for 15 min, with a 5 min resting period between games. For the purposes of this study, the other user was always a member of the study team with experience performing the virtual exercises, in a situation akin to the expert user employed in an aforementioned study [29]. This individual was located in a different room within the same building to simulate home use.

The HEP therapy consisted of pre-defined sets of seated, self-paced arm-hand exercises derived from standard care. These 16 exercises were presented to the participant in the form of a printed handout (see Additional file 1). The HEP consisted of a generalized list of tasks for the specific purposes of this research study, and not one individualized to the needs of a particular patient. In accordance with the other modalities, we instructed participants to work through the handout in three blocks of 15 min of activity followed by 5 min of rest for each training session. Participants performed all tasks while seated at a table. Roughly 30% of the exercises involved arm support (provided by the tabletop) while 70% were unsupported. To limit the potential for participants to perform extra HEP at home, no copies of the HEP protocol were permitted to leave the research facility.

**Fig. 3:**
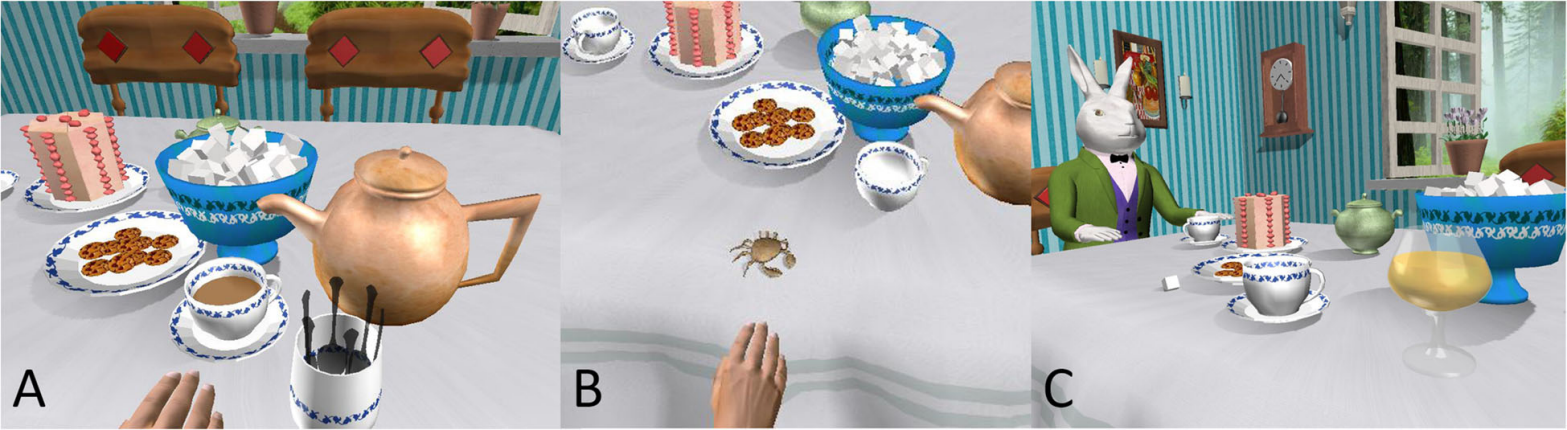
Three AWVR exercises used for this study. First-person user view is shown. **a** *Tea Stir*. Spoons (foreground) melt when used to stir the hot tea. **b** *Crabby Cookies*. Cookies morph into crabs that run around the table and must be “captured”. **c** *Bottomless Sherry.* Sherry glass must be repeatedly lifted to “drink” and set down on the table to refill

The AWVR is a rich VRE on which we have trained stroke survivors in the past [39]. The environment draws the user into the March Hare’s tea party where the participant is guided to perform a number of tasks to spur repetitive practice of movements. We chose three of the available exercises, based on their appropriateness for encouraging arm movement. One exercise, *Tea Stir*, entailed picking up a spoon from a jar and then reaching forward to stir tea in a teacup; the arm remained suspended, unsupported until the spoon “melted” and disappeared (Fig. 3a). This exercise involved crossing the midline of the body and extending the arm laterally. For the *Crabby Cookies* exercise, the participant reached out to a plate of virtual cookies. Once a cookie was touched, it transformed into a crab that scurried across the table and had to be “caught” by touching it (Fig. 3b). Thus, the *Crabby Cookies* exercise required multiple reaches away from the body. The third exercise, *Bottomless Sherry*, required the participant to reach for a glass of sherry, raise it to take a sip, and then set it back on the table to be refilled (Fig. 3c). We instructed participants to place the glass in different locations on the surface of the table to encourage reaching to her range of motion limits. All exercises were performed with the arm unsupported. Participants performed each exercise for 15 min, followed by a 5 min break, over the one-hour session. As the complexity was similar across exercises, order of play was random and often chosen by the participant.

During the first therapy session for each therapy modality, study staff explained the exercises and demonstrated performance. After that, study staff were available throughout each session to answer questions and monitor for safety, but each participant was encouraged to work independently, as if at home. As noted for VERGE, the participant worked together with another member of the study staff, but that individual was located in a separate room.

**Fig. 4:**
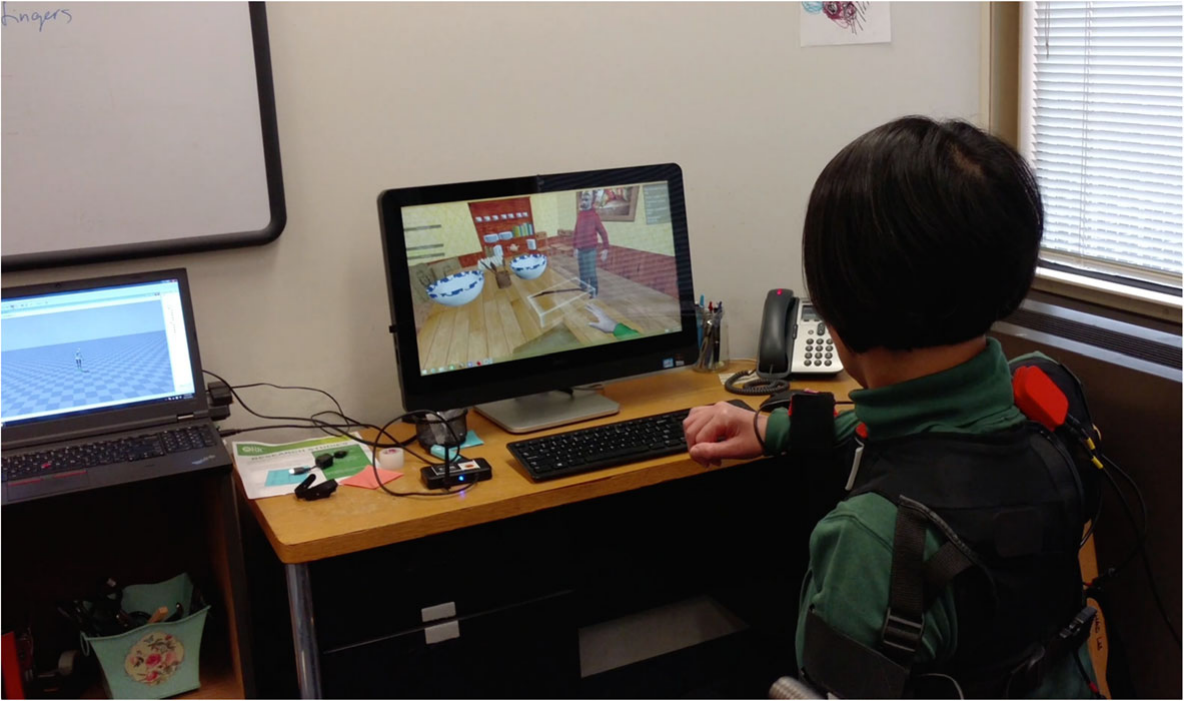
Stroke survivor training while wearing Xsens. Participant is trying to erase the displayed trajectory in the *Trajectory Trace* exercise in VERGE. While control of the avatar is provided through the Kinect™, the subject is wearing an Xsens 3D motion tracker system vest (MVN, Xsens, Culver City, CA) to provide continuous measurement of the hand and shoulder displacement for experimental analysis

#### Outcome measures

As we were especially interested in user response to VERGE, we administered a questionnaire (*VERGE Survey*) employing a 5-point Likert scale [47, 48] at the end of the VERGE training week (see Additional file 2). This questionnaire addressed the different exercises and issues specific to VERGE. Participants completed a different questionnaire (*Weekly Survey*) at the end of each therapy week (including VERGE) to measure the level of engagement in and the perceived potential for the therapy (*See* Additional file 3). We administered a final questionnaire (*Final Survey*), directly comparing the three therapies, at the end of the study (*See* Additional file 4). Additionally, we captured participant kinematics throughout the third session for each therapy modality with the Xsens 3D motion tracker system (MVN, Xsens, Culver City, CA), (Fig. 4). Participants donned the Xsens vest, containing eight inertial measurement units (IMUs) and a headband containing one IMU (per upper extremity configuration). The Xsens system continuously recorded upper extremity movement from the IMU data during all of the exercises.

#### Analysis

Descriptive statistics were utilized to compare responses from the *VERGE* Survey. For the *Weekly Survey*s, we used the nonparametric Friedman test to compare participant responses, as measured by the Likert scores, across the treatment modalities. Hand and shoulder displacements were computed from the Xsens data by using the biomechanical toolkit Mokka [49]. Total shoulder movement was subtracted from total hand movement to account for arm displacement resulting purely from trunk translation. We performed non-inferiority tests to examine whether total arm displacement during VERGE was inferior to arm displacement during the other two modalities. The δ was set equal to 10% of the larger of the hand displacement means for AWVR and HEP. Furthermore, we examined which parts of the workspace were accessed. For example, Reach Distance was calculated as the relative distance, normalized by arm length, of the hand away from the shoulder. Thus, a Reach Distance = 0 indicated that the hand was coincident with the shoulder, while a Reach Distance = 1 indicated full arm extension. We defined Hand Elevation as the vertical location of the hand with respect to the shoulder. A value of 0 represented the hand at shoulder height, while a value of 1 indicated that the hand was located at its lowest possible position with respect to the shoulder (i.e., hand at the side with elbow fully extended). We performed non-inferiority tests to examine whether time spent with a Reach Distance ≥ 0.7 and whether the time spent with Hand Elevation ≤ 0.4 were inferior for VERGE than for the other two modalities. The δ was set to 4.5 min, equal to 10% of the total training time. We also created histograms to show the amount of time the hand was positioned at different bins of Reach Distance and Hand Elevation.

## Results

### Questionnaires

User feedback gathered for the *VERGE Survey* was generally positive, with 13 of 15 participants indicating that the therapy was *Very* (*n* = 8) or *Extremely* (*n* = 5) productive and 14 of 15 participants indicating that they were *Satisfied* (*n* = 2) or *Very Satisfied* (*n* = 12) with the amount of arm use during the therapy session. Additionally, participants largely enjoyed having a partner as 13 of the 15 participants *Very Much* (*n* = 7) or *Extremely* (*n* = 6) enjoyed playing with a virtual partner and 14 of 15 participants *Agreed* (7) or *Strongly agreed* (7) that training with another virtual partner in the environment increased motivation. Response to the look of the exercises was generally positive as 11 of 15 participants *Very Much* (*n* = 6) or *Extremely* (*n* = 5) liked the 3D graphics of the system. Importantly, 12 of 15 participants *Agreed* (*n* = 5) or *Strongly Agreed* (*n* = 7) that the VERGE system had great potential as home-based rehabilitation.

Results of the *Weekly Survey*, administered at the end of each training modality, are shown in Table 1. Few statistically significant differences were apparent among the three treatment modalities for any of the responses. *Satisfaction with time spent in training* was significantly less for VERGE than for the other modalities; however, all three modalities showed a mean response above 4, which represented *Satisfied* on the survey. Responses were generally quite positive, with the vast majority of responses having a mean value of 4.0 or greater (5-point Likert scale with 3 indicating a neutral response). Importantly, the majority of subjects indicated that if the equipment were available they would *Definitely* (*n* = 10) or *Probably* (*n* = 4) continue at least one of the therapy modalities at home. Overall, they indicated a willingness to perform the therapy for 2–3 or more days per week (*n* = 15).

**Table 1:** Results for *Weekly Survey* administered at the end of week for each modality

Survey Question	VERGE	AWVR	HEP	Friedman
Importance of exercise speed	4.6 (0.6)	4.3 (1.1)	4.6 (0.7)	0.733
Importance of personal progress	4.9 (0.3)	4.5 (0.7)*	4.9 (0.4)	0.038
Importance of performance of activities of daily living	4.6 (0.6)	4.6 (0.8)	4.9 (0.4)	0.522
Importance of ability to perform new tasks	4.5 (1.0)	4.6 (0.7)	4.9 (0.3)	0.264
Importance of greater arm/hand movement	4.8 (0.4)	4.9 (0.5)	4.9 (0.4)	0.717
Level of interest/stimulation from therapy	4.0 (1.0)	4.2 (0.7)	4.1 (1.0)	0.889
Effectiveness/helpfulness for arm/hand	4.2 (0.8)	4.4 (0.6)	4.2 (1.0)	0.368
Satisfaction with ease of use	4.3 (1.0)	4.5 (0.8)	4.4 (1.0)	0.661
Satisfaction with your attention to exercises	4.6 (0.8)	4.3 (0.8)	4.7 (0.6)	0.206
Satisfaction with your desire to complete training	4.8 (0.8)	4.9 (0.4)	4.7 (0.5)	0.311
Satisfaction with progress	4.2 (0.8)	4.3 (0.7)	4.6 (0.6)	0.061
Satisfaction with amount of arm use	4.7 (0.8)	4.7 (0.5)	4.7 (0.5)	1.0
Satisfaction with time spent in training	4.1 (0.8)*	4.6 (0.5)	4.6 (0.5)	0.032
Progress experienced in training	3.7 (1.1)	3.9 (0.9)	3.5 (1.1)	0.565
Amount of arm movement compared to prior treatment	4.1 (0.8)	4.5 (0.6)	4.3 (0.8)	0.439
Continued home use	3.9 (1.4)	4.3 (0.8)	4.0 (1.4)	0.304
Expected frequency of home exercises	3.2 (1.0)	3.3 (0.7)	3.2 (1.2)	0.595
Rehabilitation potential	4.1 (1.1)	4.1 (0.9)	4.4 (0.7)	0.206

Likert scale from 1 to 5 employed. Higher numbers denote more positive responses. Mean (SD)

* indicates significance at the level indicated by the Friedman test statistic

The questionnaire directly comparing the training modalities (*Final Survey*) revealed a variety of opinions (Table 2). Participants were fairly split about which of the modalities they found the most engaging (each modality received 5 selections) and which they preferred (5 each selected HEP and AWVR and 4 selected VERGE). However, we observed strong trends favoring HEP regarding ease of understanding (two-thirds of participants selected HEP) and which therapy users would most likely continue in the home (9 of 15 participants selected HEP).

**Table 2:** Results of Final Survey comparing the three training paradigms

Characteristic	VERGE	AWVR	HEP
Most engaging	5	5	5
Greatest desire to complete sessions	3	6	6
Moved arm the most	4	4	7
Easiest to understand	1	3	10
Most effective	1	5	8
Preferred form of therapy	4	5	5
Most likely to continue at home	3	3	9

Values listed reflect number of subjects choosing each modality

### Kinematics

**Fig. 5:**
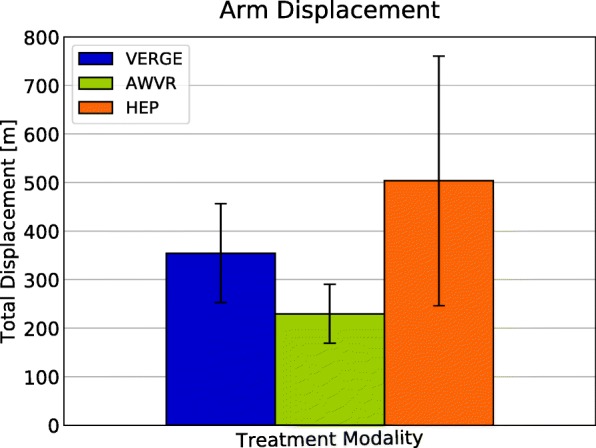
Average total arm displacement all subjects for each of the three training modalities. Movement tracked across third training session for each modality. Error bar represents one standard deviation

**Fig. 6:**
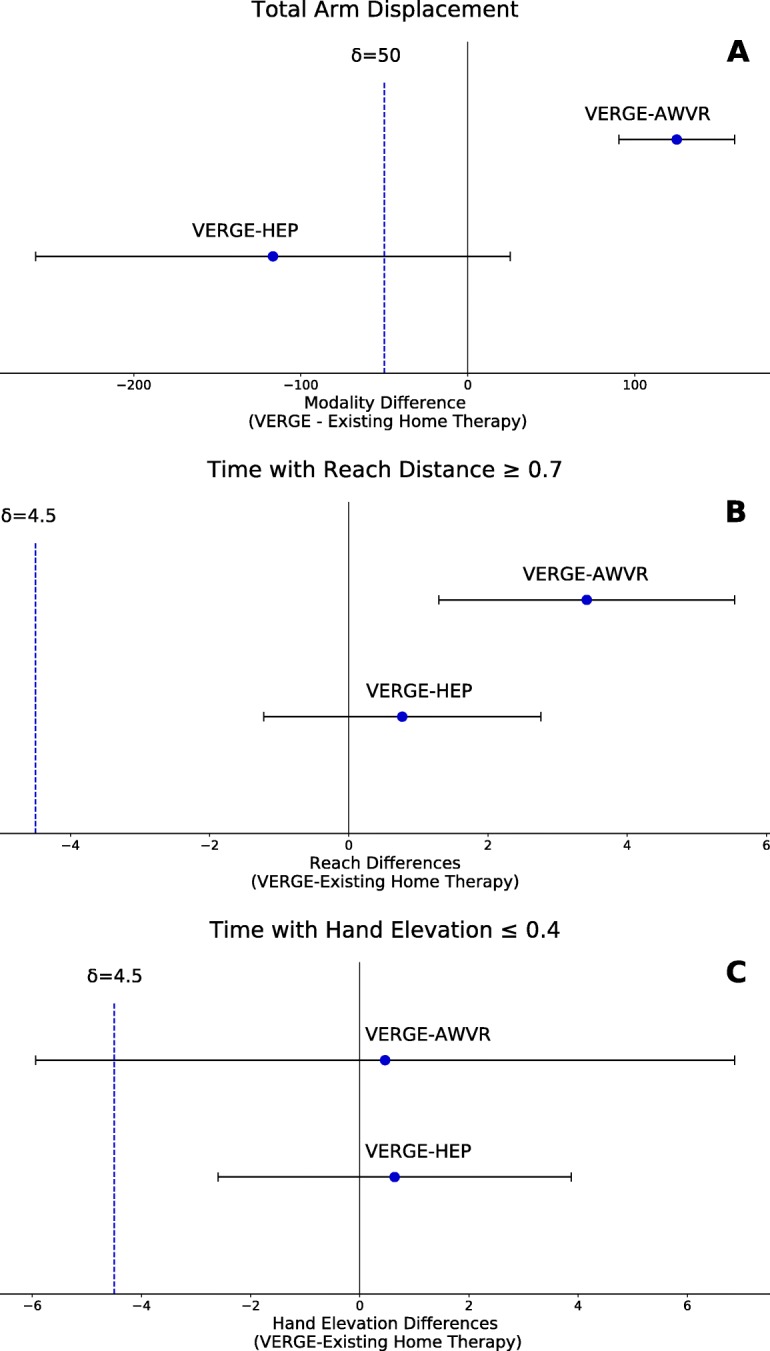
Non-inferiority tests. **a** Total arm displacement. The δ was set equal to 10% of HEP mean. **b** Time spent (in minutes) with Reach Distance greater than or equal to 70% of arm length. **c** Time spent (in minutes) with Hand Elevation within 40% of arm length with respect to the shoulder. The δ was set equal to 10% of full training time (45 min) for **b** and **c**. VERGE not inferior to HEP or AWVR for any of these measures

**Fig. 7:**
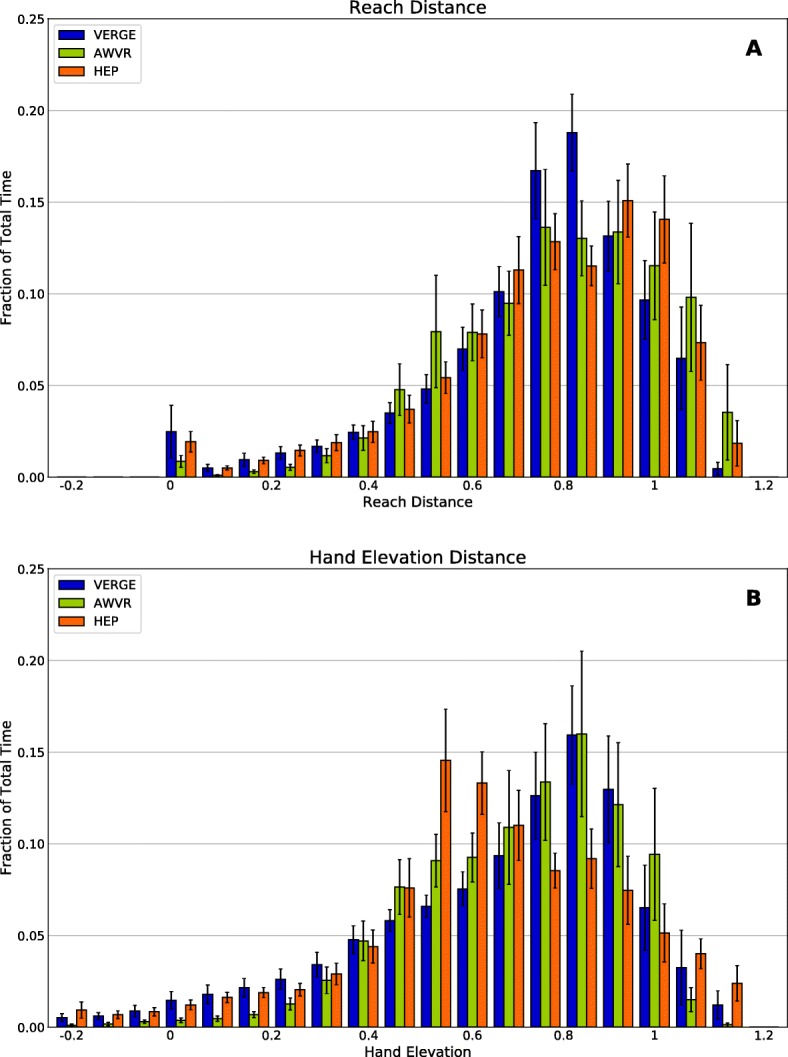
Histograms, averaged across all subjects, depicting time the hand of the impaired limb spent in different regions of the workspace. **a** Reach Distance. Distance of the hand from the shoulder, represented as a fraction of arm length (0: hand coincident with shoulder; 1: arm fully extended). **b** Hand Elevation Distance. Vertical location of the hand with respect to the shoulder, represented as a fraction of arm length (0, hand elevation equal to shoulder elevation; 1: hand elevation full arm length below shoulder elevation; negative values indicate hand elevation above shoulder elevation). Blue: VERGE, green: AWVR, orange: HEP

Each therapy modality promoted considerable arm movement. Total arm displacement averaged: 354 m for VERGE, 503 m for HEP, and 229 m for AWVR (Fig. 5). Non-inferiority testing showed that arm displacement produced by participants during the VERGE training was not significantly inferior to HEP or AWVR (Fig. 6a). All three modalities also encouraged extended arm postures and elevated hand positions. Time spent with Reach Distance ≥ 0.7 for VERGE was not inferior to that for the other two modalities (Fig. 6b). Additionally, the largest histogram values occurred for postures at which the hand was extended 70% or more of full-arm length away from the shoulder (Fig. 7a). The VERGE system also promoted elevating the hand at least as much as other modalities. Time spent with Hand Elevation ≤ 0.4 was not inferior for VERGE as compared to AWVR and HEP (Fig. 6c). The histograms revealed that during the VERGE session, subjects spent 18.2% (± 14.3%) of the time with the hand at or within 40% of arm length below shoulder height (Fig. 7b), while they spent 10.6% (± 0.9%,) with the AWVR and 16.5% (± 12.3%) during the HEP.

## Discussion

### VERGE implementation

We developed a 3D, networked VR system allowing users, physically remote from each other, to interact within a virtual environment. Each user controls an avatar in real time by movement of corresponding body segments. These avatars can manipulate virtual objects located within the environment; multiple avatars can even manipulate the same object, such as a ball hit back and forth. Each user needs only have a computer, wireless mouse, and a Kinect™ device. No special software is required for the user, only an executable version of our code and the Kinect SDK.

This VERGE system was successfully tested by 15 stroke survivors with chronic hemiparesis in the upper extremity. User response was generally positive, with 85% of the participants expressing satisfaction with the utility of the therapy and 93% indicating satisfaction with the amount of arm movement induced. Indeed, participants moved their hands an average of 350 m (after subtracting shoulder translation) during each session. This far exceeds the amount of hand displacement produced by the 54 movements observed during a typical occupational therapy session [50]. In accordance with previous multi-user training studies [26, 29, 30], the vast majority (14 of 15) participants indicated that they liked having a partner for therapy, despite not being in visual contact with this person.

### Comparison with other potential training modalities

Overall, all three therapy options encouraged considerable movement of the hand in space. Non-inferiority testing confirmed that use of the VERGE system did not result in significantly less displacement of the hand than that recorded using the more established AWVR or HEP modalities. As relatively few studies have quantified arm movement during therapy outside of a robotic device, these values provide an important target for therapy. Importantly, all three training modalities encouraged movement away from the body. Arm movements to areas of the workspace which require elbow extension can be challenging for stroke survivors, especially when the arm is unsupported [51, 52], as was the case for VERGE, AWVR, and the majority of HEP. The differences observed in the amount of arm movement between modalities could be a result of confounding factors in exercise design. Specifically, exercises in VERGE were designed to include movements out of synergy and in a large free 3-D space. Although the importance of 3D movements in therapy is a topic of debate [34, 35], many tasks require non-planar movements. VERGE allows practice of such task-based motions. AWVR also included movements out of synergy but the workspace was much more limited in size. HEP included many exercises with proximal arm stabilization, these movements were simpler in that they did not require multiple joint coordination or trunk stabilization.

During the training sessions, participants spent the most time with their arms extended at least 70% of full range. Participants also spent a considerable portion of time with the hand raised in an upper level of the workspace (within 40% of arm length of the shoulder elevation). With VERGE, for example, participants spent almost 20% of the session with their hand in this region of the workspace despite not having arm support.

Users, however, indicated differences in experience across the treatment modalities. While participants rated the modalities similarly in the weekly questionnaires, they expressed a preference for the HEP in certain areas in the comparative questionnaire, including as the most effective therapy and the treatment they would most likely continue in the home. Some of the appeal is undoubtedly attributable to the ease of use. Two-thirds of participants chose HEP as the easiest to use. This needs to be addressed in VERGE, as we describe in the following section.

### Limitations and lessons learned

The study identified limitations with VERGE that need to be addressed for improved acceptance and utility. For example, the *Trajectory Trace* exercise placed a significant cognitive demand on users. They were required to actively cycle through a sequence of discrete states for each round (Draw, Claim, Trace, Reset) while coordinating with another player (i.e., one player would draw a trajectory while another would claim and trace it). It was sometimes difficult to determine the current state and to remember which came next. Thus, while almost equal numbers of subjects listed *Ball Bump* or *Food Fight* as their favorite VERGE exercise, none listed *Trajectory Trace.* Despite similar amounts of time spent on each VERGE exercise, hand displacement during *Trajectory Trace* was less than 50% of the amount seen during the *Ball Bump* exercise. Partially attributable to this, over 70% of participants chose HEP as the easiest to understand (only one subject picked VERGE). Clearly, reducing complexity of operation for the user of a therapy paradigm is of paramount importance. We have subsequently modified *Trajectory Trace* to include a visual display indicating the state flow and current state.

Due to the largely collaborative nature of the tasks in VERGE, they included limited quantitative performance measures for the users. Participants stressed the need for objective feedback. As noticeable functional changes may evolve slowly, quantitative assessment of game performance, which can show gains on a much shorter timescale, may provide the motivation needed in the short-term to enable reaching functional milestones. We have subsequently added scoring for each of the exercises. In some cases, both competitive and collaborative scoring is available.

There were limitations with the pilot study as well. While one of the potential benefits of the VERGE system is the inclusion of other players, we did not directly examine preferences for individual vs. partnered training, as previous studies have done [26, 27, 29, 30]. In our study, the three training modalities were quite different from each other. The HEP and AWVR represented existing modalities with potential for use in the home. Factors such as ease of use, the engaging nature of the virtual task, or scoring undoubtedly influenced preferences for the chosen training modality. It should be noted that a large majority of participants expressed enthusiasm for playing with a partner when using VERGE and indicated that they felt that the presence of another user increased motivation. Enthusiasm for multiple players may have been even greater if a friend or relative had served as the playing partner, as was the case in a previous study [26].

Our relatively small sample of participants displayed considerable motivation in repeatedly coming to the laboratory in the hospital for the study and maintaining study adherence. The enthusiasm for therapy may not be as great in the general population. Additionally, only three training sessions were performed with each modality. User responses may have been different after more sessions.

User response may also have been impacted by the fact that this pilot study was performed in the laboratory rather than the home. Coming to the hospital, interacting with people, and receiving compensation may have elevated interest, particularly for the HEP. Compliance rates for conventional home therapy exercise programs have been mixed [53–56].

## Conclusions

This represents one of the first tests of stroke survivors interacting with a remote user in a 3D virtual environment for therapy. The VERGE system can be directly utilized for home-based therapy with a family member or friend in their home or a therapist in the clinic. The low cost and minimal requirements make it practical for the clinic or home. Most participants expressed satisfaction with the system and enthusiasm for the virtual partner. However, they did stress the importance of ease of use and feedback of performance. Their responses highlighted the need for technology to be sufficiently flexible to accommodate the different goals and preferences of individual users.

Importantly, participants indicated a strong interest in home therapy. Over 66% responded that they would *Definitely* be willing to continue therapy in the home and 100% responded that they would perform the training at least 2–3 times per week. Two-thirds of participants indicated that they would be willing to perform home-based training 6–7 times per week. While limitations must be addressed, multi-user virtual reality environments hold promise for maintaining engagement in therapy and providing feedback of performance for home users. We are currently undertaking a home therapy study with the VERGE system.

## Additional files

Additional file 1: Home Exercise Program (HEP) handout. The HEP consisted of pre-defined sets of seated, self-paced arm-hand exercises derived from the standard of care. These 16 exercises were presented to the participant in the form of a printed handout. (PDF 261 kb)

Additional file 2: VERGE Survey. Questionnaire administered following the VERGE therapy addressing system specific details. (PDF 28 kb)

Additional file 3: Weekly Survey. Questionnaire administered weekly following each therapy modality (including VERGE). (PDF 311 kb)

Additional file 4: Final Survey. Questionnaire administered at the end of the study to compare the three treatment modalities. (PDF 30 kb)

## Abbreviations

ANOVA: Analysis of variance
AWVR: Alice in Wonderland virtual reality
CMSA: Chedoke-McMaster stroke assessment
HEP: Home Exercise Program
ROM: Range of motion
SDK: Software development kit
TCP: Transmission Control Protocol
UDP: User Datagram Protocol
VERGE: Virtual Environment for Rehabilitative Gaming Exercises
VR: Virtual reality
