# Supplementary material for: Development of a 3D, networked multi-user virtual reality environment for home therapy after stroke
Source: J Neuroeng Rehabil. 2018 Oct 5;15:88. doi: 10.1186/s12984-018-0429-0 (PMC6173932; doi:10.1186/s12984-018-0429-0)
Supplement: Supplementary file 1 — Home Exercise Program (HEP) handout. The HEP consisted of pre-defined sets of seated, self-paced arm-hand exercises derived from the standard of care. These 16 exercises were presented to the participant in the form of a printed handout. (PDF 261 kb) [file 12984_2018_429_MOESM1_ESM.pdf]

Tabletop Exercises

Position	Exercises	Amount
**Away from the table**		
	**Shoulder Shrug – Curious Turtle**<br>Shrug BOTH of your shoulders up and down<br>• Pull those shoulders down as far away from your ears as possible!	10x
	**Chest out**<br>Pull both of your shoulders back<br>• Squeeze your shoulder blades together<br>• Try to Touch your elbows together behind your back<br>• Keep your shoulders DOWN away from your ears	10x
	**Butterfly**<br>1. Bend your elbows to 90*,<br>2. Pull elbows away from your body<br>3. Then pull them elbows in tight to your body<br>• Like you're 'flapping your wings'<br>• Keep your shoulders down away from your ears<br>• Sit up straight, don't lean back	10x
	**Hand to mouth**<br>Move your hand from your lap to your mouth<br>• Focus on bending your elbow as much as possible<br>• If you can't reach your mouth, go as far as you can	10x
	**Marching Soldier**<br>Let your arm hang at your side, try to raise your arm directly in front of you<br>• Keep your elbow as straight as possible<br>• Keep your body still<br>• Lead with your thumb if possible<br>• Use a weight if it's too easy	10x

At the table	Too easy? Do these with wrist weights on.	
Directly facing table<br><br>Washcloth under hand	**Wipe the table:**<br>Put your hand flat on the table. Trace LINE #1<br>• Keep your body still<br>• Just move your arm	10x
45* to table<br><br>Washcloth under elbow (optional)	**Lift Hand**<br>Put your elbow on the table (on the "x"), then lift your hand as high as you can<br>• Keep your elbow on the table, don't let it come up!<br>• Keep your body as still as possible<br>• Too easy? Try palm facing up (toward your face)	10x
Directly facing table<br><br>Washcloth under hand	**Place your bet**<br>Push your hand forward as far as you can, and then you're your hand back to the edge of the table<br>• Trace LINE #2<br>• Keep your body still, just move your arm<br>• Try to straighten your elbow as much as possible	10x
45* to table<br><br>Elbow on the "x"<br><br>Foam in hand	**Ready to eat**<br>1. Start with the fork resting on the table<br>2. Turn it upward so it is straight up and down (perpendicular to the table)<br>3. Return it SLOWLY to resting flat on the table.<br>• Keep your body still<br>• Too easy?<br>o Use an umbrella<br>o Turn the fork all the way (180 degrees)	10x
45* to table<br><br>Cup in hand	**Thirsty**<br>Bring the cup to your mouth<br>• With elbow on "x", keeping it on table throughout movement<br>• Away from the table, starting with your hand in your lap<br>• Try to keep the cup up and down as if you're going to drink from a straw.	- 10x with elbow on "x"<br><br>-10x with hand in lap
Facing table directly	**How fast?**<br>Reach your hand to each tape dot, in order (1-10)<br>Now do this again as quickly as you can. Use the stopwatch in your other hand and write down your time.	-10x warm up<br><br>-10x as fast as possible!

<table>
<tr><td></td><td colspan="2">Theraband Exercises</td><td></td></tr>
<tr><td>Position:</td><td>- Sit in a chair
- Feet flat
- Back and hips to the back of the chair</td><td>- Wrap theraband around both hands
- If it’s too easy use red theraband (yellow is easier)</td><td></td></tr>
<tr><td></td><td colspan="2">Pull up
- Hold your unaffected hand in your lap, keep it there
- Pull up with your affected hand
- Keep your elbow as straight as you can</td><td>10x</td></tr>
<tr><td></td><td colspan="2">Pull out
- Start with both hands in the center
- Pull both hands to the side
- Make the theraband stretch as long as possible!</td><td>10x</td></tr>
<tr><td></td><td colspan="2">Start the lawn mower
1. Start with both hands in your lap
2. Lead with your elbow, hand palm face down
3. Pull up and across your body like you are starting a lawn mower
- Keep you hand close to your chest
- Pull your shoulder blade back
- Stick your chest out!
- Your elbow should be the highest part of your arm</td><td>10x</td></tr>
</table>
