# Supplementary material for: Development of a 3D, networked multi-user virtual reality environment for home therapy after stroke
Source: J Neuroeng Rehabil. 2018 Oct 5;15:88. doi: 10.1186/s12984-018-0429-0 (PMC6173932; doi:10.1186/s12984-018-0429-0)
Supplement: Supplementary file 2 — VERGE Survey. Questionnaire administered following the VERGE therapy addressing system specific details. (PDF 28 kb) [file 12984_2018_429_MOESM2_ESM.pdf]

VERGE Survey

The following survey is about your experience with the Multi-user virtual reality game only. Please circle the best answer for each of the following questions.

1. For the multi-user VR, which exercise did you prefer?

☐ Ball game	☐ Tracing	☐ Food fight

Why? (Please specify using 3 keywords)

_________________ _________________ _________________

2. For the multi-user VR, how helpful did you find the audio connection option?

Extremely Very Somewhat Not really Not at all

3. For the multi-user VR, did you enjoy having the other player present?

Extremely Very Somewhat Not really Not at all

4. For the multi-user VR, how much did you like the graphics of the game 3D environment?

Extremely Very Somewhat Not really Not at all

5. For the multi-user VR, would you enjoy the game more if the 3D kitchen environment would be an exact replica of your own kitchen?

Extremely Very Somewhat Not really Not at all

6. Would you recommend any other exercises?

____

____

For Questions 7 and 8, how much do you agree with the following statements?

7. “It would be great if this rehabilitation program was accessible from my home 24/7.”

Strongly Agree	Agree	Neither Agree Nor Disagree	Disagree	Strongly Disagree

8. “I have better motivation for my training sessions if I train with my virtual partner rather then alone”

Strongly Agree	Agree	Neither Agree Nor Disagree	Disagree	Strongly Disagree

Thank you!
