# Supplementary material for: Development of a 3D, networked multi-user virtual reality environment for home therapy after stroke
Source: J Neuroeng Rehabil. 2018 Oct 5;15:88. doi: 10.1186/s12984-018-0429-0 (PMC6173932; doi:10.1186/s12984-018-0429-0)
Supplement: Supplementary file 3 — Weekly Survey. Questionnaire administered weekly following each therapy modality (including VERGE). (PDF 311 kb) [file 12984_2018_429_MOESM3_ESM.pdf]

Please take a few moments to complete this questionnaire. Feel free to ask for clarification when answering any of the items. Your honest answers and comments to all questions will be very helpful to our research team as we develop rehabilitation programs.

1. Which type of therapy did you participate in this week? Circle your choice.

   Home Exercise Program (HEP)

   Alice in Wonderland VR (AWVR)

   Virtual Environment for Rehabilitative Gaming Exercises (VERGE)

2. From your perspective, how important are the following outcomes in your rehabilitation training?

	Very Important	Important	Neutral	Somewhat Important	Not Important
Ability to do the exercises faster					
Sense of personal progress					
Better performance of activities of daily living (e.g. getting dressed, eating,)					
Ability to do new tasks					
Greater arm/hand movement					
Other, please explain in the space below					

____
____
____

3. Using three words or short phrases, describe your experience using this program:

____________________ ____________________ ____________________

4. How interesting or stimulating did you find this therapy?

   ☐ Extremely ☐ Very ☐ Somewhat ☐ Not really ☐ Not at all

5. How helpful did you find this therapy in for using your arm and hand?

   ☐ Extremely ☐ Very ☐ Somewhat ☐ Not really ☐ Not at all

6. How can we make this therapy better?

____

____

____

7. When you think about the therapy you had this week, how satisfied are you with:

	Very Satisfied	Satisfied	Neither Satisfied nor Dissatisfied	Dissatisfied	Very Dissatisfied
Ease of use					
How well it kept your attention					
Your desire to complete training					
Your sense of progress					
Amount of arm use during therapy					
Time spent in therapy					
Other, please explain in the space below					

____

____

____

For questions 8 and 9, compare **<u>1 week</u>** of this therapy to **<u>1 week</u>** of other types of therapy you received for your arm in the past.

8. How much progress did you experience during this therapy?

   Much More More Same Less Much Less

9. How much did you move your arm this therapy?

Much More | More | Same | Less | Much Less

10. Would you choose to continue this form of therapy at home?

Definitely | Probably | Not Sure | Probably Not | Definitely Not

11. How often do you think you would complete these exercises at home?

6-7 times/week | 4-5 times/week | 2-3times/week | Once/week | Never

12. What would limit you from doing all recommended training sessions at home with this therapy (check all that apply)?

- ☐ Nothing – I would do them
- ☐ Too complicated to play
- ☐ Too boring
- ☐ Too frustrating
- ☐ I am too busy
- ☐ I would forget to do them
- ☐ I would rather do something else
- ☐ Other (please specify) ________________________________

13. Based on your experience, please rate the rehabilitation potential you see in this type of therapy:

Excellent | Good | Moderate | Small | None

14. Please share ideas you have to improve this treatment.

____
____
____

15. Please share any further thoughts about this rehabilitation experience using keywords or short phrases.

____
____
____

Thank you!
