# Supplementary material for: Development of a 3D, networked multi-user virtual reality environment for home therapy after stroke
Source: J Neuroeng Rehabil. 2018 Oct 5;15:88. doi: 10.1186/s12984-018-0429-0 (PMC6173932; doi:10.1186/s12984-018-0429-0)
Supplement: Supplementary file 4 — Final Survey. Questionnaire administered at the end of the study to compare the three treatment modalities. (PDF 30 kb) [file 12984_2018_429_MOESM4_ESM.pdf]

Final Survey

Please circle the type of therapy that best answers each of the following questions.

HEP – Home Exercise Program
AWVR – Alice in Wonderland Virtual Reality
VERGE – Virtual Environment for Rehabilitative Gaming Exercises

1. Which therapy did you find to be the **most engaging**?
   ☐ HEP ☐ AWVR ☐ VERGE

2. For which therapy did you have the greatest **desire to complete the entire session**?
   ☐ HEP ☐ AWVR ☐ VERGE

3. For which therapy did you feel you **moved your arm the most**?
   ☐ HEP ☐ AWVR ☐ VERGE

4. For which therapy were the exercises **easiest to understand**?
   ☐ HEP ☐ AWVR ☐ VERGE

5. Which therapy did you feel was the **most effective**?
   ☐ HEP ☐ AWVR ☐ VERGE

6. Which therapy did you **prefer**?
   ☐ HEP ☐ AWVR ☐ VERGE

7. Which therapy would you be **most likely to continue at home**?
   ☐ HEP ☐ AWVR ☐ VERGE

8. For each task in the table below, which therapy helped you the most to be able to do the activity better at home (using both hands)?

**Task**	**HEP**	**AWVR**	**VERGE**	**N/A**
Washing/drying dishes				
Sweeping the floor				
Folding laundry				
Putting on a shirt				
Eating meals				
Brushing teeth				

Thank you!
